# Antifungal Potential of the Skin Microbiota of Hibernating Big Brown Bats (*Eptesicus fuscus*) Infected With the Causal Agent of White-Nose Syndrome

**DOI:** 10.3389/fmicb.2020.01776

**Published:** 2020-07-23

**Authors:** Virginie Lemieux-Labonté, Nicole A. S.-Y. Dorville, Craig K. R. Willis, François-Joseph Lapointe

**Affiliations:** ^1^Département de Sciences Biologiques, Université de Montréal, Montréal, QC, Canada; ^2^Department of Biology, Centre for Forest Interdisciplinary Research, The University of Winnipeg, Winnipeg, MB, Canada

**Keywords:** fungal infection, microbiota, resistance, big brown bat, 16S rRNA gene

## Abstract

Little is known about skin microbiota in the context of the disease white-nose syndrome (WNS), caused by the fungus *Pseudogymnoascus destructans* (*Pd*), that has caused enormous declines of hibernating North American bats over the past decade. Interestingly, some hibernating species, such as the big brown bat (*Eptesicus fuscus*), appear resistant to the disease and their skin microbiota could play a role. However, a comprehensive analysis of the skin microbiota of *E. fuscus* in the context of *Pd* has not been done. In January 2017, we captured hibernating *E. fuscus*, sampled their skin microbiota, and inoculated them with *Pd* or sham inoculum. We allowed the bats to hibernate in the lab under controlled conditions for 11 weeks and then sampled their skin microbiota to test the following hypotheses: (1) *Pd* infection would not disrupt the skin microbiota of *Pd*-resistant *E. fuscus*; and (2) microbial taxa with antifungal properties would be abundant both before and after inoculation with *Pd*. Using high-throughput 16S rRNA gene sequencing, we discovered that beta diversity of *Pd*-inoculated bats changed more over time than that of sham-inoculated bats. Still, the most abundant taxa in the community were stable throughout the experiment. Among the most abundant taxa, *Pseudomonas* and *Rhodococcus* are known for antifungal potential against *Pd* and other fungi. Thus, in contrast to hypothesis 1, *Pd* infection destabilized the skin microbiota but consistent with hypothesis 2, bacteria with known antifungal properties remained abundant and stable on the skin. This study is the first to provide a comprehensive survey of skin microbiota of *E. fuscus*, suggesting potential associations between the bat skin microbiota and resistance to the *Pd* infection and WNS. These results set the stage for future studies to characterize microbiota gene expression, better understand mechanisms of resistance to WNS, and help develop conservation strategies.

## Introduction

The skin is the first physical and immunological barrier against invading pathogens. It is also a complex and dynamic ecosystem inhabited by a rich community of microorganisms composed of bacteria, archaea, fungi, and viruses. This community contributes to host defenses by limiting colonization and persistence of pathogens that compete for resources, and by producing pathogen inhibitors (Grice and Segre, 2011; Walter et al., 2018; Woo et al., 2019). Here, we use the term ‘microbiota’ to refer to the taxonomic diversity of Bacteria and Archaea assessed using marker genes, rather than ‘microbiome,’ which refers to both taxonomic and functional diversity of the complete community (Marchesi and Ravel, 2015). The microbiota also interact with the innate and adaptive immune system and contribute to maintenance of skin integrity and tissue repair (Lai et al., 2009; Curtis and Sperandio, 2011; Naik et al., 2012). Despite this importance, the complex interactions among the skin microbiota, skin invading pathogens and hosts remain poorly documented in animals, especially for newly emerged wildlife diseases causing population declines around the globe (Daszak, 2000; Belden and Harris, 2007; Jones et al., 2008; Fisher et al., 2012). Introduced fungal pathogens such as *Batrachochytrium dendrobatidis* and *Batrachochytrium salamandrivorans* have caused global declines and even extinctions in amphibians (Longcore et al., 1999; Martel et al., 2013). In recent decades, studies have highlighted the potential role of skin microbiota in patterns of resistance and susceptibility to these fungal pathogens and have pointed to potential management practices that might help conserve host populations (Harris et al., 2009; Bletz et al., 2013, 2017; Jani and Briggs, 2014; Woodhams et al., 2014; Bataille et al., 2016; Ange-Stark et al., 2019; Rebollar et al., 2019). However, despite this potential, management actions based on the skin microbiota have still not been widely applied in response to wildlife disease.

Similar to amphibians, hibernating North American bats have suffered dramatic impacts from a newly introduced fungal disease, white-nose syndrome (WNS) (Blehert et al., 2009; Frick et al., 2016). WNS is caused by the fungus *Pseudogymnoascus destructans* (*Pd*) (Gargas et al., 2009; Lorch et al., 2011) and has caused widespread declines of hibernating bats since being introduced from Eurasia sometime around 2006 (Warnecke et al., 2012; Frick et al., 2015, 2017; Hoyt et al., 2016; Drees et al., 2017; Trivedi et al., 2017). In vulnerable bat species, *Pd* invades the skin creating lesions that alter fluid balance, thermoregulation, and gas exchange (Meteyer et al., 2009; Cryan et al., 2010; Warnecke et al., 2012; Verant et al., 2014; McGuire et al., 2017). Hibernating bats survive the winter using stored body fat and prolonged energy-saving bouts of torpor characterized by dramatically reduced body temperature and metabolism (Jonasson and Willis, 2012; Czenze et al., 2013; Czenze and Willis, 2015). The fungal infection causes bats to warm up too frequently which, in turn, depletes their fat reserves (Reeder et al., 2012; Warnecke et al., 2012). Understanding how bat species respond to this disease is important for designing management strategies to protect bat populations both for biodiversity conservation, and preservation of the ecosystem services that bats provide (Boyles et al., 2011; Maine and Boyles, 2015; Wray et al., 2018). There is enormous variation in the impacts of WNS for different bat species, with some exhibiting little to no mortality, and others facing local extinction (Frick et al., 2010, 2015; Langwig et al., 2015, 2017). Mechanisms underlying this variation in susceptibility are not fully understood, despite potential benefits for disease management. Variation in susceptibility to *Pd* could reflect mechanisms that allow for resistance to infection (i.e., reduction or elimination of pathogen infection) and/or tolerance of infection (i.e., reduction of the harm caused by infection) (Råberg et al., 2007, 2009; Svensson and Råberg, 2010) and potential mechanisms of resistance and tolerance are beginning to be addressed in the WNS context (Frick et al., 2016; Langwig et al., 2017; Cheng et al., 2019).

Big brown bats (*Eptesicus fuscus*) exhibit evidence of resistance with mild WNS symptoms compared to more susceptible species (Frank et al., 2014; Moore et al., 2018). *E. fuscus* are of particular interest as they often hibernate under similar environmental conditions as species that are highly vulnerable to WNS, including the little brown bat (*Myotis lucifugus*), the tricolored bat (*Perimyotis subflavus*), and the northern long-eared bat (*Myotis septentrionalis*), all three of which are now listed as endangered in Canada due to WNS (Canadian Wildlife Service and Committee on the Status of Endangered Wildlife in Canada, 2013). Physiological and behavioral factors could play a role in this resistance (Willis and Wilcox, 2014; Langwig et al., 2015; Frick et al., 2016) and understanding these mechanisms could help fulfill an important knowledge gap by identifying traits that contribute to WNS survival.

One underexplored factor that could affect resistance to WNS is the skin microbiota. Differences in lipid profiles affecting WNS-resistance (Frank et al., 2016, 2018) may contribute to different microbial profiles by providing different nutritional substrates. Hundreds of microorganisms isolated from wild bats and their corresponding habitats have been tested in controlled laboratory conditions for their inhibitory effects on *Pd*, by focusing on the actions of secreted compounds, contact inhibition or volatile molecules (Hamm et al., 2017; Micalizzi et al., 2017). Little brown bats persisting with *Pd* have proportionally more abundant *Rhodococcus* and *Pseudomonas* in their skin microbiota (Lemieux-Labonté et al., 2017), suggesting a possible role for these microorganisms in bat survival. Moreover, antifungal strains of *Pseudomonas* isolated from the skin of *E. fuscus* inhibit *Pd* growth *in vitro* (Hoyt et al., 2015; Hamm et al., 2017), and improve survival of WNS-infected little brown bats when applied as a probiotic treatment *in vivo* (Cheng et al., 2017; Hoyt et al., 2019). All of these results are promising but they do not provide a complete picture of the complex skin community and its potential role in *Pd* resistance of some species.

We explored the skin microbiota of WNS-resistant *E. fuscus* inoculated with *Pd*. This species can hibernate under the same environmental conditions as more susceptible species like *M. lucifugus* but survives *Pd* infection with limited skin colonization (Walke et al., 2015; Moore et al., 2018). Therefore, *E. fuscus* represents a good model to study potential resistance mechanisms resulting from the skin microbiota in a controlled environment that accounts for variation in hibernation conditions. We tested two hypotheses about the skin microbiota of *E. fuscus* in the context of *Pd* infection: (1) that *Pd* inoculation is not a strong selective force on the skin microbiota of this species (Ange-Stark et al., 2019) and would therefore not disrupt the microbial community, and (2) that microbial taxa with antifungal properties, which are common in the hibernation sites of bats persisting after WNS (e.g., *Rhodococcus* and *Pseudomonas*, Lemieux-Labonté et al., 2017), would be abundant both before and after infection with *Pd* in this resistant species.

## Materials and Methods

### Collection of Bats

On January 18, 2017, we visited Richard Lake Mine, a hibernaculum approximately 100 km east of Kenora, ON, Canada (49°45′N, −94°28′W), which houses several hundred *M. lucifugus* and *E. fuscus* each winter. We first swabbed eight *E. fuscus* in the cave to test whether transport to the laboratory affected the skin microbiota (Supplementary Files 1, 2). We then collected 32 adult *E. fuscus* (16 males and 16 females), suspended them in cloth bags in a cooler lined with wet towels to maintain hibernation conditions, and transported them by car to a bio-secure animal facility at the University of Winnipeg. All handling of bats in the lab occurred in a biosafety cabinet. We swabbed the left wing of each torpid bat immediately after arrival in the lab to sample the “pre-captivity” hibernation microbiota. We swabbed the dorsal surface of the left wing (forearm and under forearm) in linear strokes for 20 s with a sterile Whatman Omniswab (Fisher Scientific) soaked in sterile 0.15 M NaCl (Lemieux-Labonté et al., 2017). Swabs tips were ejected into MoBio Powersoil DNA isolation Kit tubes (Mo Bio Laboratories), which were then transferred to 4°C within 2 h and to −20°C within 12 h of sampling. As a negative control, a humidified sterile swab was exposed to open air for 20 s, prior to ejecting its tip into a MoBio tube. We analyzed the skin microbiota between the inoculated and control groups at the beginning of the experiment to detect any pre-existing differences occurring by chance. After collecting the microbiota swab we then swabbed the right wing to determine *Pd* status.

All individuals were then randomly assigned to one of 4 cages with 8 individuals/cage in a 1:1 sex ratio (i.e., 4 males and 4 females/cage). We replicated the experiment in two incubators with one cage of *Pd*-inoculated bats, and one cage of sham-inoculated controls per incubator. Incubators were maintained at 8°C and 98% relative humidity. *Pd*-inoculated bats received a dose of *Pd* that has repeatedly been shown to cause symptoms of WNS in wild bats: 20 μl of inoculum, containing 500,000 conidia suspended in phosphate buffered saline (PBS) with Tween 20 to prevent clumping, pipetted onto the ventral side of their wings (Lorch et al., 2011; Warnecke et al., 2012; McGuire et al., 2016). Healthy controls were inoculated only with PBS-Tween 20 (Warnecke et al., 2012; McGuire et al., 2016).

After 11 weeks of captive hibernation, bats were swabbed again, as described above, to assess the “post-captivity” skin microbiota (left wing) and *Pd* load (right wing). We recorded body temperature immediately after removing bats from the incubator at the end of the study using a digital thermometer accurate to ±0.1°C (SPER Scientific, Model 80008, Arizona, United States) and inserting a 1 mm diameter l-type thermocouple probe 4 mm into the rectum (Supplementary File 3). Bats were then swabbed within ∼1–2 min of body temperature measurement. Only a maximum of 2–3 min would have elapsed from the time bats started rewarming in response to our disturbance until the time we swabbed them. After obtaining additional measurements and samples for a range of complementary studies, bats were humanely euthanized by CO_2_ inhalation under isoflurane anesthesia.

*Pd* swabs were processed and analyzed at the Pathogen and Microbiome Institute at the Northern Arizona University, whereas skin microbiota samples were shipped to Université de Montréal (Québec, Canada) for further processing. Areas of the wing that are infected and colonized by *Pd* hyphae fluorescence orange under UV lightning (Turner et al., 2014; Cheng et al., 2017). We took UV photographs of every bat’s wings using a digital camera (Olympus^©^ Tough TG-830) using a Canadian dime as a sizing reference, and then digitally quantified these areas as a measure of *Pd* load and infection intensity using the Image-J^©^ software. All methods were approved by the University of Winnipeg Animal Care Committee (Protocol Number AEO08399).

### DNA Extraction, Amplification and Sequencing

Bacterial genomic DNA was extracted from each swab using the MoBio Powersoil DNA isolation kit. We followed the manufacturer’s protocol, modified following Castelino et al. (2017), by adding a 15-min incubation period at 70°C after the addition of buffer C1 to increase the efficiency of microbial cell lysis. All procedures were conducted in a laminar flow hood to limit potential sample contamination, and extractions were randomized to avoid detecting false patterns (Salter et al., 2014). Four extraction blanks, two amplification blanks, and the HM-782D Human Microbiome Project mock community (BEI Resources) were also included to detect possible contamination and assess sequencing accuracy (Salter et al., 2014; Glassing et al., 2016). Because of contamination and low input DNA, 23 pre- and post-captivity *E. fuscus* skin microbiota samples, and five sampling negative controls were retained for subsequent analysis. Amplification and sequencing were performed as previously described (Preheim et al., 2013a; Lemieux-Labonté et al., 2017). Briefly, libraries were prepared using a two-step PCR. The first PCR amplified the hypervariable region V4 of the 16S small subunit ribosomal gene with forward primer U515 F and reverse primer E786 R (Caporaso et al., 2011). The amplifications were performed with a Mastercycler Nexus GSX1 (Eppendorf). Each sample was amplified in quadruplicate and pooled to limit possible PCR artifacts. The second PCR step consisted of adding primers containing a barcode (index) and Illumina adapter sequences to each DNA amplicon, and used forward primer PE-III-PCR-F and reverse primer PE-III-PCR-001-096 (Preheim et al., 2013b). This second amplification was performed in triplicate. After each PCR step, samples were pooled and purified with the PCR purification Agencourt AMPure XP (Beckman Coulter). Indexed samples concentration was measured with Qubit 2.0 Fluorometer (Invitrogen), and samples were pooled to obtain a final concentration range between 10 and 20 ng/μl. DNA was next diluted and denatured according to the manufacturer’s protocol for paired-end sequencing using MiSeq Reagent Kit v2 (500 cycles) 2 × 250 bp on MiSeq (Illumina).

### Data Analysis

For all sequences, quality filtering, trimming, dereplication, sample inference and merging of paired-end sequences, were performed in DADA2 version 1.14.1 (Callahan et al., 2016). The corresponding amplicon sequence variants (ASV) table providing ASV abundances was assigned in DADA2 using SILVA database release 132 (Quast et al., 2012).

We amplified 8,282,605 sequences classified into 43,436 ASV from the 67 samples sequenced. We filtered out mitochondrial and chloroplastic DNA sequences, as well as sequences from the genera *Halomonas* and *Shewanella*, the two most abundant taxa in negative controls. ASVs with abundance values smaller than 10 were filtered out leaving 5,176,773 sequences in 17,162 ASVs. After these filtration steps, only a small number of sequences (less than 11,000) were identified in the sampling control, extraction and PCR negative controls, and these samples were also excluded. One sampling negative control had 47,608 sequences, but it was excluded from our analysis because it diverged dramatically in composition from bat samples collected (see Supplementary File 4). A total of 5,098,811 sequences, classified in 17,162 ASVs, were amplified and analyzed from the 46 bat samples, with a mean of 110,843 sequences per sample (range: 34,416–300,474). We were able to match all expected genera in the mock positive control (see Supplementary File 5). The genera of the 20 expected mock taxa were the most abundant in the mock profile (see Supplementary File 5). We detected 733 false positives, but with very low relative abundance values (<0.1%). All analyses were conducted in R version 3.5.0 (R Core Team, 2018).

### Alpha Diversity

We first quantified diversity of the skin microbial community for all samples based on alpha diversity using the Shannon index (Shannon, 1948). The Shannon index, which includes both ASVs richness and evenness, was selected due to its reduced sensitivity to sample depth differences (Haegeman et al., 2013; Preheim et al., 2013a) (see Supplementary File 6). Normally distributed alpha diversity values were compared using linear models and linear mixed-effect models [*lm()* and *lme()* function] in R with Shannon index as the response variable, *Pd*-inoculation versus control (hereafter ‘inoculation’) and pre- versus post-captivity (hereafter ‘captivity’) as fixed effects and cage ID as a random effect. Significance was tested using ANOVA for linear model and a likelihood ratio test with a chi-square distribution for linear mixed-effect models (Pinheiro et al., 2017).

### Beta Diversity

We used two distance measures to account for the phylogeny of microbiota in our samples (i.e., unweighted UniFrac and weighted UniFrac) (Lozupone and Knight, 2005; Lozupone et al., 2007). Distances were computed on rarefied data, as such measures could be sensitive to differences in sequencing depth (Lozupone et al., 2011; Weiss et al., 2017). Computations were performed with the *phyloseq* package (McMurdie and Holmes, 2013) and results were visualized with principal coordinates analysis (PCoA) (Gower, 1966) using the *ordinate()* function in R. Distance matrices were checked with the function *is.euclid()* of the *ade4* package (Dray and Dufour, 2007) prior to the ordination to ensure that all distances were Euclidian and properly representable by PCoA (Gower and Legendre, 1986). When required, square-root transformations were applied to obtain distance matrices satisfying the Euclidian condition (e.g., Weighted UniFrac). All phylogeny-based UniFrac distances were calculated using a phylogenetic tree constructed with FastTree 2.1.8 (Price et al., 2010).

To test for effects of inoculation and captivity we used distance-based redundancy analysis (db-RDA) (Legendre and Anderson, 1999). It is computed by first decomposing UniFrac distances (weighted or unweighted) into principal coordinates, and then applying RDA to the corresponding principal coordinates using the *capscale()* function of the R package *vegan* (Oksanen et al., 2019). We computed partial db-RDA to better understand the influence of *Pd*-inoculation and captivity on variation in microbial assemblages while controlling for possible confounding factors (i.e., incubator, sex, and cage ID) (Davies and Tso, 1982). Adjusted *R*-squared values (Ezekiel, 1930) were calculated to compare the explanatory power of different models. Significance of db-RDA and partial db-RDA was tested via 9999 permutations with the *anova.cca()* function of the R package *vegan*. We then performed an analysis of multivariate homogeneity (PERMDISP) (Anderson, 2006) with the *betadisper()* function to test whether groups differed in their dispersion. The null hypothesis of this test is that the average within-group dispersion is identical in all groups (Anderson, 2001). For these two tests, the number of permutations was set to 9999. To test for an effect of inoculation on any change in the microbiota from pre- to post-captivity, we calculated distances between paired pre- and post-captivity samples for each individual, and then used linear mixed effects models to test for the fixed effect of inoculation on this distance while controlling for cage ID as a random effect (Pinheiro et al., 2017). For all analyses, a *p*-value threshold of 0.05 was considered significant.

### Analysis of Skin Microbiota Composition

We assessed the effect of inoculation and captivity on the composition of microbial taxa down to the genus level using the Analysis of Composition of Microbiomes (ANCOM 2.0) (Mandal et al., 2015). ANCOM is based on non-parametric tests (i.e., either Kruskal–Wallis test for independent samples, or Friedman test for dependent samples) and is appropriate for compositional data (Gloor et al., 2017). The test relies on point estimates of data transformed by an additive log ratio, where presumed invariant taxa are selected as the denominator. To identify differences between samples, the analysis was performed using an unrarified ASVs table only including taxa with a relative abundance larger than or equal to 0.1%. The relative composition of skin microbiota was assessed using the same ASVs table.

## Results

### *Pd* Status and Bat Data

We selected Richard Lake Mine for our study, in part because, based on our surveillance in winter 2016 (swabs from bats and substrates) and fall 2016 (swabs from swarming bats), it was negative for *Pd* the year before this study. It was also >350 km from the nearest known WNS-positive hibernaculum at the time of our study (U.S. Geological Survey, 2019) and we observed no signs of WNS at the time of capture. We swabbed bats to confirm *Pd*-negative status as soon as we returned to the lab after capture but it was not possible to wait for result for these swabs before assigning bats to experimental groups. Unfortunately, after we began the experiment, qPCR results revealed that eight of the 23 study animals selected for microbiota analysis were just above the threshold (40 *C*_t_) to be considered *Pd* positive (McGuire et al., 2016) at the time of collection (39.3 ± 0.74 *C*_t_, mean ± SD; Table 1 and Supplementary File 7). Half of these eight bats had been randomly assigned to the inoculated group and half to the control group. Among the bats assigned to the control group, three *Pd*-negative bats remained negative throughout the experiment but four bats that started in the control group as negative were positive by the end of the experiment presumably because of transfer of *Pd* from naturally infected control bats. One control bat that was *Pd*-positive at the time of capture was *Pd*-negative by the end of the experiment (Table 1). We do not think this infection of captured bats, or contamination of the control group, influenced our results because loads at capture were extremely low, near the limit of detection and, on average, 18-fold lower than loads for the bats we experimentally infected (see Supplementary File 7). Moreover, proportions of the wings exhibiting orange UV fluorescence (see Supplementary File 7) of contaminated control bats at the end of the experiment (0.2 ± 0.4%) were 14-fold lower than those of inoculated bats (2.8 ± 3.3%) (Wilcox test, *W* = 6, *p* < 0.001) and *Pd* loads of inoculated bats (0.0003 ± 0.0003 ng) were six-fold that of the contaminated control bats at the end of the experiment (0.00005 ± 0.0001 ng) (Wilcox test, *W* = 24, *p* = 0.01). All bats that we inoculated with *Pd* in the laboratory, and used for subsequent analysis of the microbiota (*n* = 12), were *Pd*-positive at the end of the experiment.

**TABLE 1:** Information from *E. fuscus* individuals sampled skin microbiota.

**Sample ID**	**Inoculation^1^**	**Incubator**	**Sex**	**Pd status qPCR Pre**	**Pd loads Pre (ng)**	**Pd status qPCR Post**	**Pd loads Post (ng)**	**Fluorescence Post**
EPFU11	PBST	2	Female	Negative	0	Positive	0.0000553	Nothing
EPFU13	PBST	1	Female	Negative	0	Negative	0	Nothing
EPFU15	PBST	1	Female	Negative	0	Positive	0.0000331	Nothing
EPFU18	PBST	2	Male	Negative	0	Positive	0.000045	Nothing
EPFU19	PBST	1	Male	Negative	0	Negative	0	Blue
EPFU24	PBST	1	Male	Negative	0	Negative	0	Blue
EPFU26	PBST	2	Female	Positive	0.0000052	Positive	0.0000604	Blue
EPFU30	PBST	2	Male	Positive	0.0000052	Positive	0.0000305	Orange–blue
EPFU31	PBST	1	Male	Positive	0.0000059	Negative	0	Orange–blue
EPFU4	PBST	2	Female	Positive	0.0000406	Positive	0.000375	Orange–blue–green
EPFU7	PBST	1	Female	Negative	0	Positive	0.0000053	Orange–blue
EPFU1	*Pd*	1	Female	Negative	0	Positive	0.000389	Orange–blue
EPFU10	*Pd*	1	Female	Positive	0.000006	Positive	0.0007221	Orange–blue–green
EPFU12	*Pd*	2	Male	Negative	0	Positive	0.0000695	Orange–blue
EPFU16	*Pd*	1	Female	Negative	0	Positive	0.0000948	Orange–blue
EPFU2	*Pd*	2	Female	Negative	0	Positive	0.0007946	Orange–blue–green
EPFU20	*Pd*	1	Male	Negative	0	Positive	0.0002084	Orange
EPFU23	*Pd*	1	Male	Positive	0.0000067	Positive	0.0001392	Orange–green
EPFU27	*Pd*	1	Male	Negative	0	Positive	0.0000109	Orange
EPFU29	*Pd*	2	Male	Negative	0	Positive	0.0000446	Orange
EPFU3	*Pd*	2	Female	Positive	0.0000057	Positive	0.0000425	Orange–blue–green
EPFU32	*Pd*	2	Male	Positive	0.0000051	Positive	0.0006427	Orange–green
EPFU5	*Pd*	2	Female	Negative	0	Positive	0.0000098	Orange

*^1^Pd, Pd inoculated bats; PBST, sham inoculated bats PBS + 0.5%Tween 20.*

After experiment, we detected orange fluorescence typical of *Pd* infection on *E. fuscus* skin of all bats we inoculated and on four control bats (Table 1). Interestingly, green and/or blue fluorescence was also detected. Green was always detected with co-occurrence of orange and/or blue fluorescence, whereas blue fluorescence was detected in the presence of orange, or alone.

### Alpha Diversity

After assuring no difference in Shannon diversity between inoculation groups prior to the experiment (*F* = 0.62, *p* = 0.44), no effect of inoculation was detected on Shannon diversity at the end of the experiment while controlling for cage ID (χ2 = 0.05, *p* = 0.82). There was also no effect of inoculation on the change in alpha diversity from capture to the end of the experiment (*F* = 0.74, *p* = 0.40). Although there was no effect of inoculation, we did detect a significant difference in alpha diversity between the start and the end of the experiment (*F* = 93.15, *p* < 0.0001). Mean Shannon diversity values decreased by 24% (1.59, ± = 0.16) in post-inoculated samples indicating a strong effect of either hibernation or captivity (or both) on alpha diversity of the skin microbiota.

### Beta Diversity

By chance, the inoculation group factor explained redundant variation in weighted UniFrac distances when we controlled for sex pre-captivity (Table 2). However, this redundant variation was not persistent throughout the experiment. Indeed, there was no redundant variation between inoculation and UniFrac distances by the end of the experiment (i.e., for post-captivity samples) after controlling for incubator, sex and cage ID (Table 2). This indicates that beta diversity changed over the course of the experiment, although it was not caused by inoculation. Yet, cage ID was the factor that explained most of the variation in beta diversity when included in the model post-captivity (Table 2). Dispersion was homogeneous according to inoculation in pre-captivity samples (PERMDISP unweighted UniFrac, *F* = 0.87, *p* = 0.36; PERMDISP weighted UniFrac *F* (3.15, p = 0.09) and in post-captivity sample (PERMDISP unweighted UniFrac, *F* = 2.23, *p* = 0.16; PERMDISP weighted UniFrac, *F* = 1.12, *p* = 0.30).

**TABLE 2:** db-RDA of unweighted and weighted UniFrac distances of *E. fuscus* skin microbiota samples.

**Distance measure**	**Model**	***F* statistic**	**Adjusted *R*^2^**	***P*-value**
Unweighted UniFrac	Captivity timepoint	16.70	0.26	**0.0001**
	Inoculation pre	0.99	NA	0.46
	Inoculation post	0	NA	0
	Cage ID post	2.24	0.06	**0.0017**
Weighted UniFrac	Captivity timepoint	29.54	0.42	**0.0001**
	Inoculation pre	0	NA	0
	Inoculation post	0	NA	0
	Cage ID post	2.72	0.08	**0.02**

*Captivity timepoint model test for difference between pre- and post-captivity samples while sex factor is controlled. Inoculation pre model test for difference between treatment group pre-captivity while controlling sex. Inoculation post model test for difference between treatment group post-captivity while controlling incubator, sex and cage ID. Cage ID post model test for difference in cage post-captivity while controlling incubator, sex and inoculation. Significant results are in bold.*

**FIGURE 1:**
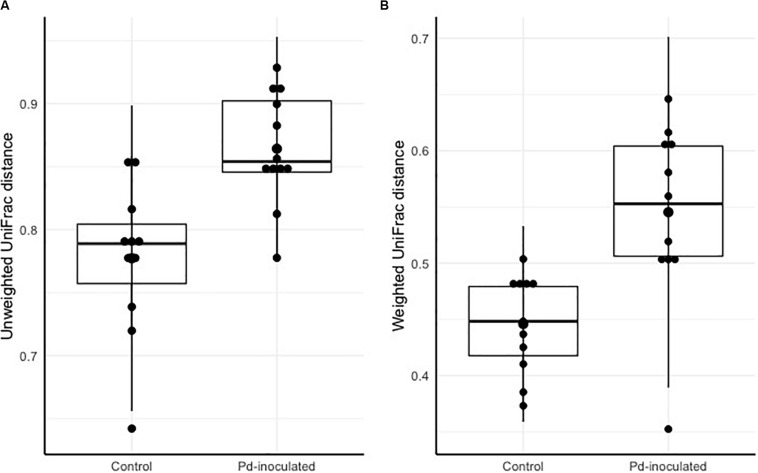
Pre- and post-captivity paired distance grouped by inoculation. **(A)** Pre-and post-captivity paired Unweighted UniFrac distances grouped by inoculation. **(B)** Pre-and post-captivity paired Weighted UniFrac distances grouped by inoculation. The black bars represent standard deviations, and the black points represent mean alpha diversity values.

To account for temporal effects and better assess whether inoculation led to more or less change in the microbiota over the course of the experiment, we calculated distances between pre- and post-captivity samples paired within each individual. After controlling for cage ID, we found significantly higher dispersion of distances for *Pd*-inoculated bats based on both unweighted and weighted UniFrac distances (χ^2^ = 6.52, *p* = 0.01, χ^2^ = 8.92, *p* = 0.003) (Figure 1). Post-captivity samples from inoculated bats were 11% (0.09 ± 0.03) more distant than samples from controls based on unweighted UniFrac distances, and 22% (0.10 ± 0.03) more distant based on weighted UniFrac (Figure 1). In other words, as for pooled results above, within individuals the skin microbiota of Pd-inoculated bats changed more over time than that of sham-inoculated controls.

**FIGURE 2:**
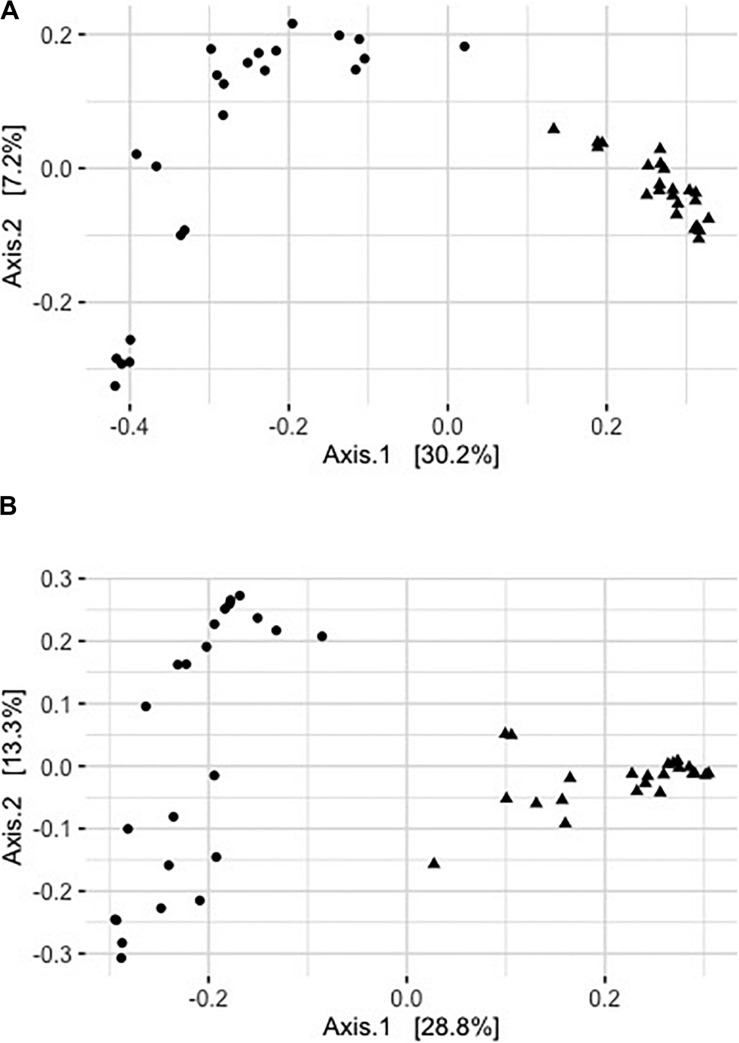
Principal coordinates analysis of rarefied pre- (triangles) and post-captive (circles) bat samples (36,416 sequences). **(A)** Principal coordinate analysis of unweighted UniFrac distances. **(B)** Principal coordinate analysis of square rooted weighted UniFrac distances. Each point represents a sample from an individual bat.

Captivity was an important factor shaping community structure of the skin microbiota (Figure 2). About 26% of the variation in community structure was explained by captivity based on unweighted UniFrac distances, and about 42% of this variation was explained by captivity based on weighted UniFrac (Table 2). Community structure of pre-captivity samples was more dispersed than that in post-inoculated samples based on unweighted UniFrac distances (PERMDISP, *F* = 12.34, *p* = 0.0008) and weighted UniFrac (PERMDISP, *F* = 30.33, *p* = 0.0001) (Figure 2).

### Analysis of Skin Microbiota Composition

Only one taxon (i.e., *Pseudonocardia*) changed in abundance over the course of captivity at the genus level and it was more abundant in pre-captive individuals (Table 3). No taxa were significantly more abundant in *Pd*-inoculated individuals at the end of the experiment (Table 3).

**TABLE 3:** Differently abundant taxa detected with Analysis of Composition of Microbiomes (ANCOM) comparing paired bat pre- and post-captivity and comparing post-captive bats according to inoculation.

**Comparison**	**Taxa**	***W* statistic**
**Captivity timepoint**	**Pseudonocardia**	**12**
	Staphylococcus	10
	Hafnia.Obesumbacterium	10
	Arthrobacter	10
	Rhodococcus	10
	Pseudarthrobacter	10
	Bacillus	9
	Mycoplasma	9
	Klebsiella	8
	Actinobacillus	8
	Clostridium	8
	Pseudomonas	7
	Yersinia	7
	Flavobacterium	5

**Post inoculation**	Pseudarthrobacter	10
	Flavobacterium	9
	Pseudomonas	8
	Arthrobacter	7
	Clostridium	7
	Klebsiella	5
	Rhodococcus	5
	Pseudonocardia	4
	Staphylococcus	4
	Bacillus	4
	Actinobacillus	4
	Hafnia.Obesumbacterium	3
	Yersinia	1
	Mycoplasma	0

*Analysis was performed on unrarefied table at genus level with relative abundance higher or equal to 0.1%. Significant taxa obtained p < 0.05 after taxa-wise multiple correction. Significant results are in bold.*

**FIGURE 3:**
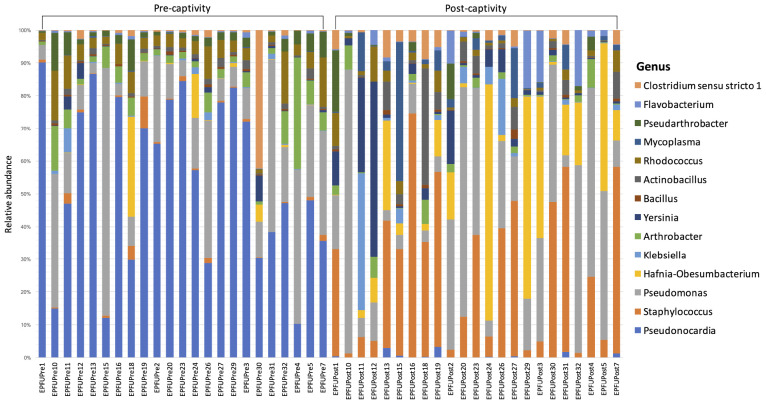
Relative abundance of different genera in the microbiota on bat skin from before (pre-captivity) and after (post-captivity) experiment. Analysis was performed on unrarefied ASVs table of taxa with relative abundance higher or equal to 0.1%.

Consistent with these results, community composition of the microbiota remained similar over the course of captivity and only a higher proportion of *Pseudonocardia* was detected in pre-captivity samples (Table 3 and Figure 3). The dominant genera we observed in the skin microbiota of *E. fuscus* were *Mycoplasma*, *Pseudomonas*, *Staphylococcus*, *Hafnia.Obesumbacterium*, *Pseudonocardia*, *Pseudarthrobacter*, *Arthrobacter*, *Bacillus*, *Klebsiella*, *Actinobacillus*, *Clostridium*, *Yersinia*, *Flavobacterium*, and *Rhodococcus*. Based on ANCOM results (Table 3) *Pseudomonas*, *Mycoplasma*, *Staphylococcus*, *Hafnia.Obesumbacterium*, *Pseudarthrobacter*, *Arthrobacter*, *Bacillus*, *Klebsiella*, *Actinobacillus*, *Clostridium*, *Yersinia*, *Flavobacterium*, and *Rhodococcus* appeared to remain constant over the course of captivity.

## Discussion

Research on the skin microbiota of wildlife facing emergent skin diseases is a growing area of research (Daszak, 2000; Belden and Harris, 2007; Jones et al., 2008; Fisher et al., 2012), and in the specific context of North American bats, understanding responses of the skin microbiota could contribute to management of WNS (Cheng et al., 2017; Hoyt et al., 2019). We inoculated, or sham inoculated *E. fuscus* with *Pd* and maintained them under controlled conditions for 3 months to test whether the skin microbiota of a WNS-resistant species might evolve in response to fungal infection, and help understand the potential contribution of the microbiota to WNS resistance.

We found mixed support for our first hypothesis that *Pd* inoculation would have little impact on the skin microbiota of this WNS-resistant species. No taxa were significantly more abundant on *Pd*-inoculated bats compared to controls, at the end of the experiment, and no difference was detected in diversity as observed in the wild for this species (Ange-Stark et al., 2019). Interestingly, the observed differences in beta diversity (weighted UniFrac distances) between bats assigned to our two experimental groups disappeared by the end of the experiment. This suggests that inoculation has no effect on the skin microbiota diversity. However, our results indicate that *Pd* inoculation reduced the capacity of the host (or its microbiota) to regulate the skin microbiota, leading to more dispersion within and between individuals as observed in studies of *Bd*, the fungal pathogen affecting amphibians (Zaneveld et al., 2017). Jani and Briggs (2014) found that outbreaks of *Bd* increased temporal changes in the skin microbiota of *Rana sierra* and suggested that, even frogs that could tolerate *Bd* infection could be sensitive to disruption of the microbiota by the pathogen, although whether this disruption was protective or harmful was not clear. Similarly, our results reflect a transformation of the skin microbiota due to *Pd* infection, although we do not know if this disruption would be advantageous, detrimental, or neutral for infected *E. fuscus*. In *Lithobates catesbieanus*, an amphibian species with low susceptibility to *Bd* (Eskew et al., 2015), the fungus was a selective force on the microbial community, but the microbiota also affected *Bd* infectivity and, consequently, probably exhibited a negative effect on host fitness (Walke et al., 2015). Our results could not establish whether the skin microbiota affects the fungal infection, or if the fungal infection could in turn affect the microbiota, and this still needs to be investigated.

Consistent with our second hypothesis, the composition of the microbiota included several taxa known to inhibit *Pd* growth *in vitro*, and which have been found on bats persisting after WNS [e.g., *Rhodococcus* (Cornelison et al., 2014; Hamm et al., 2017), *Pseudomonas* (Hoyt et al., 2015)]. *Pseudonocardia* was also the most abundant microbial taxa in pre-captivity samples and, although it declined in abundance by the end of the experiment, it is known to have antifungal activity (Sen et al., 2009). The proportional abundances of *Rhodococcus* and *Pseudomonas* were stable from pre- to post-captivity and they were among the most abundant taxa at both time points. These bacteria may play a role in the persistence of some individuals of highly susceptible bat species (e.g., *M. lucifugus*) after *Pd* invasion (Lemieux-Labonté et al., 2017) and the fact that they represented a large proportion of the microbiota in the resistant species we studied also suggests their importance in the response of bats to WNS. Interestingly, the blue and green fluorescence we detected on *E. fuscus* skin, is typical of pyocyanine and pyoverdine pigments, which have been observed in some *Pseudomonas* such as *Pseudomonas aeruginosa* and *Pseudomonas fluorescens* (Reyes et al., 1981; Albesa et al., 1985). Pyoverdine is a molecule that scavenges iron from the environment and could compete with pathogens such as *Pd* that may be limited by iron availability (Mascuch et al., 2015; Reeder et al., 2017; Sass et al., 2017). Pyocyanine is an exotoxin with antimicrobial potential and could also be detrimental to *Pd* (Hassan and Fridovich, 1980; Baron and Rowe, 1981). Green fluorescence (typical of pyoverdine) was always observed co-occurring with orange fluorescence (typical of *Pd*) and/or blue fluorescence (typical of pyocyanine), whereas blue fluorescence was detected both alone or alongside orange fluorescence. These patterns are consistent with antifungal activity by *Pseudomona*s on the skin of *E. fuscus*. However, it is important to note that strains of the potential genera discussed above are not obligatorily antifungal, and that the metabarcoding method used in our study did not allow us to assess the proportion of antifungal metabolites produced as suggested by a previous study (Antony-Babu et al., 2017). The proportional overview of the community is also biased by the different copy numbers of ribosomal 16S rRNA gene present in different microorganisms (Klappenbach, 2001). Further studies should focus on the functional influence of these bacteria and other microorganisms (e.g., fungi and viruses) on the host-pathogen interaction between bats and *Pd* using approaches such as whole metagenomics sequencing.

The fact that some *E. fuscus* were, unexpectedly, already *Pd*-positive when we collected them before the experiment could have influenced our results. Since all collected bats were roosting in the same site, it is possible that all bats sampled were either carrying *Pd* at levels below our detectability threshold, or had previously been exposed. However, the facts that the mine was *Pd*-negative the year before our study, that we observed no clinical signs of WNS during our capture session, and that intensity of infection was very low for the few positive bats we did collect, suggest the cave only recently became infected with *Pd* and that impacts on our results were likely minimal.

We also found that the skin microbiota shifted during captivity. This finding supports previous results in which samples from the same bat collected over time were more different than samples from multiple bats collected at the same time and place (Kolodny et al., 2019). The decrease in microbial diversity we observed in post-captivity samples likely reflects the controlled conditions in the laboratory setting. Captivity has been observed to negatively affect community diversity in the microbiota of wild amphibians (Kueneman et al., 2016; Sabino-Pinto et al., 2016) and it has been linked to sterile conditions in captivity (Loudon et al., 2014).

Our results suggest that the skin microbiota profile of *E. fuscus* may contribute to the apparent resistance of this species to *Pd.* Antifungal taxa predominated the microbiota prior to infection and remained stable in the skin microbiota after inoculation and 3 months of hibernation. However, despite apparent resistance to WNS and the abundance of anti-fungal bacteria, *Pd* could still affect host health in this species as it was shown to create skin lesion and inflammation (Moore et al., 2018) that may impact skin barrier integrity and lead to subsequent vulnerability to other pathogens. Although certain bacterial taxa remained abundant, there was destabilization of the microbiota that could be harmful for bats and this requires further study to truly understand impacts of infection on this species. We recommend that future studies try to better characterize post-*Pd* colonization patterns and, especially, characterize functional changes resulting from these patterns. WNS resistance and tolerance may reflect a range of mechanisms, from storage of extra body fat in fall (Cheng et al., 2019), endogenous fat in epidermis composition (Frank et al., 2016, 2018), variation in individual behavior (Frick et al., 2016) to variation in immune response (Maslo and Fefferman, 2015). Our results suggest that the skin microbiota could be another mechanism helping some bats survive infection with *Pd*. We recommend further research on gene expression by the microbiome of resistant and susceptible bat species to help understand disease mechanisms in WNS and contribute to conservation strategies for North American bats.

## Data Availability Statement

The datasets supporting the conclusions of this article are available in the Figshare repository. Raw data are available at https://figshare. com/s/a1015374c5bccd9f9dc7. Scripts and matrix are available at https://figshare.com/s/87d08697f1d32aeebb86.

## Ethics Statement

The animal study was reviewed and approved by The University of Winnipeg Animal Care Committee (Protocol Number AEO08399).

## Author Contributions

VL-L carried out experiments, analyzed the data, and wrote the manuscript. CW and ND designed the experiments, performed sampling, and wrote the manuscript. F-JL, VL-L, CW, and ND wrote the manuscript. All authors read and approved the final manuscript.

## Conflict of Interest

The authors declare that the research was conducted in the absence of any commercial or financial relationships that could be construed as a potential conflict of interest.

## References

[B1] Albesa I. Barberis L. I. Pajaro M. C. Eraso A. J. (1985). Pyoverdine production by *Pseudomonas* fluorescens in synthetic media with various sources of nitrogen. *Microbiology* 131 3251–3254. 10.1099/00221287-131-12-3251

[B2] Anderson M. J. (2001). A new method for non-parametric multivariate analysis of variance: non-parametric manova for ecology. *Austral. Ecol.* 26 32–46. 10.1111/j.1442-9993.2001.01070.pp.x

[B3] Anderson M. J. (2006). Distance-based tests for homogeneity of multivariate dispersions. *Biometrics* 62 245–253. 10.1111/j.1541-0420.2005.00440.x 16542252

[B4] Ange-Stark M. A. Cheng T. L. Hoyt J. R. Langwig K. E. Parise K. L. Frick W. F. (2019). White-nose syndrome restructures bat skin microbiomes. *bioRxiv* [Preprint]. 10.1101/614842 PMC10714735 37888992

[B5] Antony-Babu S. Stien D. Eparvier V. Parrot D. Tomasi S. Suzuki M. T. (2017). Multiple *Streptomyces* species with distinct secondary metabolomes have identical 16S rRNA gene sequences. *Sci. Rep.* 7:11089. 10.1038/s41598-017-11363-1 PMC5593946 28894255

[B6] Baron S. S. Rowe J. J. (1981). Antibiotic action of pyocyanin. *Antimicrob. Agents Chemother.* 20 814–820. 10.1128/AAC.20.6.814 6798928 PMC181804

[B7] Bataille A. Lee-Cruz L. Tripathi B. Kim H. Waldman B. (2016). Microbiome variation across amphibian skin regions: implications for Chytridiomycosis mitigation efforts. *Microb. Ecol.* 71 221–232. 10.1007/s00248-015-0653-0 26271741

[B8] Belden L. K. Harris R. N. (2007). Infectious diseases in wildlife: the community ecology context. *Front. Ecol. Environ.* 5 533–539. 10.1890/060122 16828735

[B9] Blehert D. S. Hicks A. C. Behr M. Meteyer C. U. Berlowski-Zier B. M. Buckles E. L. (2009). Bat white-nose syndrome: an emerging fungal pathogen? *Science* 323 227–227. 10.1126/science.1163874 18974316

[B10] Bletz M. C. Loudon A. H. Becker M. H. Bell S. C. Woodhams D. C. Minbiole K. P. C. (2013). Mitigating amphibian chytridiomycosis with bioaugmentation: characteristics of effective probiotics and strategies for their selection and use. *Ecol. Lett.* 16 807–820. 10.1111/ele.12099 23452227

[B11] Bletz M. C. Perl R. G. B. Bobowski B. T. C. Japke L. M. Tebbe C. C. Dohrmann A. B. (2017). Amphibian skin microbiota exhibits temporal variation in community structure but stability of predicted Bd-inhibitory function. *ISME J.* 11 1521–1534. 10.1038/ismej.2017.41 28387770 PMC5520157

[B12] Boyles J. G. Cryan P. M. McCracken G. F. Kunz T. H. (2011). Economic importance of bats in agriculture. *Science* 332 41–42. 10.1126/science.1201366 21454775

[B13] Callahan B. J. McMurdie P. J. Rosen M. J. Han A. W. Johnson A. J. A. Holmes S. P. (2016). DADA2: high-resolution sample inference from Illumina amplicon data. *Nat. Methods* 13:581. 10.1038/nmeth.3869 27214047 PMC4927377

[B14] Canadian Wildlife Service and Committee on the Status of Endangered Wildlife in Canada (2013). *COSEWIC assessment and status report on the Little Brown Myotis (Myotis lucifugus), Northern Myotis (Myotis septentrionalis), Tri-colored Bat (Perimyotis subflavus) in Canada.* Available online at: https://central.bac-lac.gc.ca/.item?id=CW69-14-688-2014-eng&op=pdf&app=Library (accessed January 17, 2020).

[B15] Caporaso J. G. Lauber C. L. Walters W. A. Berg-Lyons D. Lozupone C. A. Turnbaugh P. J. (2011). Global patterns of 16S rRNA diversity at a depth of millions of sequences per sample. *Proc. Natl. Acad. Sci. U.S.A.* 108 4516–4522. 10.1073/pnas.1000080107 20534432 PMC3063599

[B16] Castelino M. Eyre S. Moat J. Fox G. Martin P. Ho P. (2017). Optimisation of methods for bacterial skin microbiome investigation: primer selection and comparison of the 454 versus MiSeq platform. *BMC Microbiol.* 17:23. 10.1186/s12866-017-0927-4 28109256 PMC5251215

[B17] Cheng T. L. Gerson A. Moore M. S. Reichard J. D. DeSimone J. Willis C. K. R. (2019). Higher fat stores contribute to persistence of little brown bat populations with white−nose syndrome. *J. Anim. Ecol.* 88 591–600. 10.1111/1365-2656.12954 30779125

[B18] Cheng T. L. Mayberry H. McGuire L. P. Hoyt J. R. Langwig K. E. Nguyen H. (2017). Efficacy of a probiotic bacterium to treat bats affected by the disease white-nose syndrome. *J. Appl. Ecol.* 54 701–708. 10.1111/1365-2664.12757

[B19] Cornelison C. T. Keel M. K. Gabriel K. T. Barlament C. K. Tucker T. A. Pierce G. E. (2014). A preliminary report on the contact-independent antagonism of Pseudogymnoascus destructans by *Rhodococcus* rhodochrousstrain DAP96253. *BMC Microbiol.* 14:246. 10.1186/s12866-014-0246-y 25253442 PMC4181622

[B20] Cryan P. M. Meteyer C. Boyles J. G. Blehert D. S. (2010). Wing pathology of white-nose syndrome in bats suggests life-threatening disruption of physiology. *BMC Biol.* 8:135. 10.1186/1741-7007-8-135 21070683 PMC2984388

[B21] Curtis M. M. Sperandio V. (2011). A complex relationship: the interaction among symbiotic microbes, invading pathogens, and their mammalian host. *Mucosal Immunol.* 4 133–138. 10.1038/mi.2010.89 21248724 PMC3079440

[B22] Czenze Z. J. Park A. D. Willis C. K. R. (2013). Staying cold through dinner: cold-climate bats rewarm with conspecifics but not sunset during hibernation. *J. Comp. Physiol. B* 183 859–866. 10.1007/s00360-013-0753-4 23539327

[B23] Czenze Z. J. Willis C. K. R. (2015). Warming up and shipping out: arousal and emergence timing in hibernating little brown bats (*Myotis lucifugus*). *J. Comp. Physiol. B* 185 575–586. 10.1007/s00360-015-0900-1 25809999

[B24] Daszak P. (2000). Emerging infectious diseases of wildlife– threats to biodiversity and human health. *Science* 287 443–449. 10.1126/science.287.5452.443 10642539

[B25] Davies P. T. Tso M. K.-S. (1982). Procedures for reduced-rank regression. *Appl. Stat.* 31:244 10.2307/2347998

[B26] Dray S. Dufour A.-B. (2007). The ade4 package: implementing the duality diagram for ecologists. *J. Stat. Softw.* 22 1–20. 10.18637/jss.v022.i04

[B27] Drees K. P. Lorch J. M. Puechmaille S. J. Parise K. L. Wibbelt G. Hoyt J. R. (2017). Phylogenetics of a fungal invasion: origins and widespread dispersal of white-nose syndrome. *mBio* 8:e01941-17. 10.1128/mBio.01941-17 PMC5727414 29233897

[B28] Eskew E. A. Worth S. J. Foley J. E. Todd B. D. (2015). American bullfrogs (*Lithobates catesbeianus*) resist infection by multiple isolates of *Batrachochytrium dendrobatidis*, including one implicated in wild mass mortality. *EcoHealth* 12 513–518. 10.1007/s10393-015-1035-2 26065669

[B29] Ezekiel M. (1930). *Methods of Correlation Analysis.* Oxford: Wiley.

[B30] Fisher M. C. Henk D. A. Briggs C. J. Brownstein J. S. Madoff L. C. McCraw S. L. (2012). Emerging fungal threats to animal, plant and ecosystem health. *Nature* 484 186–194. 10.1038/nature10947 22498624 PMC3821985

[B31] Frank C. L. Ingala M. R. Ravenelle R. E. Dougherty-Howard K. Wicks S. O. Herzog C. (2016). The effects of cutaneous fatty acids on the growth of *Pseudogymnoascus* destructans, the etiological agent of white-nose syndrome (WNS). *PLoS One* 11:e0153535. 10.1371/journal.pone.0153535 27070905 PMC4829186

[B32] Frank C. L. Michalski A. McDonough A. A. Rahimian M. Rudd R. J. Herzog C. (2014). The resistance of a North American bat species (*Eptesicus fuscus*) to white-nose syndrome (WNS). *PLoS One* 9:e113958. 10.1371/journal.pone.0113958 25437448 PMC4250063

[B33] Frank C. L. Sitler-Elbel K. G. Hudson A. J. Ingala M. R. (2018). The antifungal properties of epidermal fatty acid esters: insights from white-nose syndrome (WNS) in bats. *Molecules* 23:1986. 10.3390/molecules23081986 30096918 PMC6222711

[B34] Frick W. Pollock J. F. Hicks A. C. Langwig K. E. Reynolds D. S. Turner G. G. (2010). An emerging disease causes regional population collapse of a common North American bat species. *Science* 329 679–682. 10.1126/science.1188594 20689016

[B35] Frick W. F. Cheng T. L. Langwig K. E. Hoyt J. R. Janicki A. F. Parise K. L. (2017). Pathogen dynamics during invasion and establishment of white-nose syndrome explain mechanisms of host persistence. *Ecology* 98 624–631. 10.1002/ecy.1706 27992970

[B36] Frick W. F. Puechmaille S. J. Hoyt J. R. Nickel B. A. Langwig K. E. Foster J. T. (2015). Disease alters macroecological patterns of North American bats: disease alters macroecology of bats. *Glob. Ecol. Biogeogr.* 24 741–749. 10.1111/geb.12290

[B37] Frick W. F. Puechmaille S. J. Willis C. K. R. (2016). “White-nose syndrome in bats,” in *Bats in the Anthropocene: Conservation of Bats in a Changing World*, eds Voigt C. Kingston T. (Cham: Springer), 245–262.

[B38] Gargas A. Trest M. T. Christensen M. Volk T. J. Blehert D. S. (2009). *Geomyces destructans* sp. nov. associated with bat white-nose syndrome. *Mycotaxon* 108 147–154. 10.5248/108.147

[B39] Glassing A. Dowd S. E. Galandiuk S. Davis B. Chiodini R. J. (2016). Inherent bacterial DNA contamination of extraction and sequencing reagents may affect interpretation of microbiota in low bacterial biomass samples. *Gut Pathog.* 8:24. 10.1186/s13099-016-0103-7 27239228 PMC4882852

[B40] Gloor G. B. Macklaim J. M. Pawlowsky-Glahn V. Egozcue J. J. (2017). Microbiome datasets are compositional: and this is not optional. *Front. Microbiol.* 8:2224. 10.3389/fmicb.2017.02224 29187837 PMC5695134

[B41] Gower J. C. (1966). Some distance properties of latent root and vector methods used in multivariate analysis. *Biometrika* 53:325 10.2307/2333639

[B42] Gower J. C. Legendre P. (1986). Metric and Euclidean properties of dissimilarity coefficients. *J. Classif.* 3 5–48. 10.1007/BF01896809

[B43] Grice E. A. Segre J. A. (2011). The skin microbiome. *Nat. Rev. Microbiol.* 9 244–253. 10.1038/nrmicro2537 21407241 PMC3535073

[B44] Haegeman B. Hamelin J. Moriarty J. Neal P. Dushoff J. Weitz J. S. (2013). Robust estimation of microbial diversity in theory and in practice. *ISME J.* 7 1092–1101. 10.1038/ismej.2013.10 23407313 PMC3660670

[B45] Hamm P. S. Caimi N. A. Northup D. E. Valdez E. W. Buecher D. C. Dunlap C. A. (2017). Western bats as a reservoir of novel *Streptomyces* species with antifungal activity. *Appl. Environ. Microbiol.* 83:e03057-16. 10.1128/AEM.03057-16 27986729 PMC5311414

[B46] Harris R. N. Brucker R. M. Walke J. B. Becker M. H. Schwantes C. R. Flaherty D. C. (2009). Skin microbes on frogs prevent morbidity and mortality caused by a lethal skin fungus. *ISME J.* 3 818–824. 10.1038/ismej.2009.27 19322245

[B47] Hassan H. M. Fridovich I. (1980). Mechanism of the antibiotic action pyocyanine. *J. Bacteriol.* 141 156–163. 10.1128/jb.141.1.156-163.1980 6243619 PMC293551

[B48] Hoyt J. R. Cheng T. L. Langwig K. E. Hee M. M. Frick W. F. Kilpatrick A. M. (2015). Bacteria isolated from bats inhibit the growth of *Pseudogymnoascus destructans*, the causative agent of white-nose syndrome. *PLoS One* 10:e0121329. 10.1371/journal.pone.0121329 25853558 PMC4390377

[B49] Hoyt J. R. Langwig K. E. White J. P. Kaarakka H. M. Redell J. A. Parise K. L. (2019). Field trial of a probiotic bacteria to protect bats from white-nose syndrome. *Sci. Rep.* 9:9158. 10.1038/s41598-019-45453-z 31235813 PMC6591354

[B50] Hoyt J. R. Sun K. Parise K. L. Lu G. Langwig K. E. Jiang T. (2016). Widespread bat white-nose syndrome fungus, Northeastern China. *Emerg. Infect. Dis.* 22 140–142. 10.3201/eid2201.151314 26673906 PMC4698868

[B51] Jani A. J. Briggs C. J. (2014). The pathogen *Batrachochytrium dendrobatidis* disturbs the frog skin microbiome during a natural epidemic and experimental infection. *Proc. Natl. Acad. Sci. U.S.A.* 111 E5049–E5058. 10.1073/pnas.1412752111 25385615 PMC4250152

[B52] Jonasson K. A. Willis C. K. R. (2012). Hibernation energetics of free-ranging little brown bats. *J. Exp. Biol.* 215 2141–2149. 10.1242/jeb.066514 22623203

[B53] Jones K. E. Patel N. G. Levy M. A. Storeygard A. Balk D. Gittleman J. L. (2008). Global trends in emerging infectious diseases. *Nature* 451 990–993. 10.1038/nature06536 18288193 PMC5960580

[B54] Klappenbach J. A. (2001). rrndb: the ribosomal RNA operon copy number database. *Nucleic Acids Res.* 29 181–184. 10.1093/nar/29.1.181 11125085 PMC29826

[B55] Kolodny O. Weinberg M. Reshef L. Harten L. Hefetz A. Gophna U. (2019). Coordinated change at the colony level in fruit bat fur microbiomes through time. *Nat. Ecol. Evol.* 3 116–124. 10.1038/s41559-018-0731-z 30532043

[B56] Kueneman J. G. Woodhams D. C. Harris R. Archer H. M. Knight R. McKenzie V. J. (2016). Probiotic treatment restores protection against lethal fungal infection lost during amphibian captivity. *Proc. R. Soc. B Biol. Sci.* 283:20161553. 10.1098/rspb.2016.1553 27655769 PMC5046908

[B57] Lai Y. Di Nardo A. Nakatsuji T. Leichtle A. Yang Y. Cogen A. L. (2009). Commensal bacteria regulate Toll-like receptor 3–dependent inflammation after skin injury. *Nat. Med.* 15 1377–1382. 10.1038/nm.2062 19966777 PMC2880863

[B58] Langwig K. E. Frick W. F. Reynolds R. Parise K. L. Drees K. P. Hoyt J. R. (2015). Host and pathogen ecology drive the seasonal dynamics of a fungal disease, white-nose syndrome. *Proc. R. Soc. B Biol. Sci.* 282:20142335. 10.1098/rspb.2014.2335 25473016 PMC4286034

[B59] Langwig K. E. Hoyt J. R. Parise K. L. Frick W. F. Foster J. T. Kilpatrick A. M. (2017). Resistance in persisting bat populations after white-nose syndrome invasion. *Philos. Trans. R. Soc. B Biol. Sci.* 372:20160044. 10.1098/rstb.2016.0044 27920389 PMC5182440

[B60] Legendre P. Anderson M. J. (1999). Distance-based redundancy analysis: testing multispecies responses in multifactorial ecological experiments. *Ecol. Monogr.* 69 1–24. 10.1890/0012-9615(1999)069[0001:dbratm]2.0.co;2

[B61] Lemieux-Labonté V. Simard A. Willis C. K. R. Lapointe F.-J. (2017). Enrichment of beneficial bacteria in the skin microbiota of bats persisting with white-nose syndrome. *Microbiome* 5:115. 10.1186/s40168-017-0334-y 28870257 PMC5584028

[B62] Longcore J. E. Pessier A. P. Nichols D. K. (1999). *Batrachochytrium dendrobatidis* gen. et sp. nov., a chytrid pathogenic to amphibians. *Mycologia* 91 219–227. 10.1080/00275514.1999.12061011

[B63] Lorch J. M. Meteyer C. U. Behr M. J. Boyles J. G. Cryan P. M. Hicks A. C. (2011). Experimental infection of bats with *Geomyces destructans* causes white-nose syndrome. *Nature* 480 376–378. 10.1038/nature10590 22031324

[B64] Loudon A. H. Woodhams D. C. Parfrey L. W. Archer H. Knight R. McKenzie V. (2014). Microbial community dynamics and effect of environmental microbial reservoirs on red-backed salamanders (*Plethodon cinereus*). *ISME J.* 8 830–840. 10.1038/ismej.2013.200 24335825 PMC3960541

[B65] Lozupone C. Knight R. (2005). UniFrac: a new phylogenetic method for comparing microbial communities. *Appl. Environ. Microbiol.* 71 8228–8235. 10.1128/AEM.71.12.8228-8235.2005 16332807 PMC1317376

[B66] Lozupone C. Lladser M. E. Knights D. Stombaugh J. Knight R. (2011). UniFrac: an effective distance metric for microbial community comparison. *ISME J.* 5 169–172. 10.1038/ismej.2010.133 20827291 PMC3105689

[B67] Lozupone C. A. Hamady M. Kelley S. T. Knight R. (2007). Quantitative and qualitative diversity measures lead to different insights into factors that structure microbial communities. *Appl. Environ. Microbiol.* 73 1576–1585. 10.1128/AEM.01996-06 17220268 PMC1828774

[B68] Maine J. J. Boyles J. G. (2015). Bats initiate vital agroecological interactions in corn. *Proc. Natl. Acad. Sci. U.S.A.* 112 12438–12443. 10.1073/pnas.1505413112 26371304 PMC4603461

[B69] Mandal S. Van Treuren W. White R. A. Eggesbø M. Knight R. Peddada S. D. (2015). Analysis of composition of microbiomes: a novel method for studying microbial composition. *Microb. Ecol. Health Dis.* 26:27663. 10.3402/mehd.v26.27663 26028277 PMC4450248

[B70] Marchesi J. R. Ravel J. (2015). The vocabulary of microbiome research: a proposal. *Microbiome* 3:s40168-015-0094-5. 10.1186/s40168-015-0094-5 26229597 PMC4520061

[B71] Martel A. Spitzen-van der Sluijs A. Blooi M. Bert W. Ducatelle R. Fisher M. C. (2013). *Batrachochytrium salamandrivorans* sp. nov. causes lethal chytridiomycosis in amphibians. *Proc. Natl. Acad. Sci. U.S.A.* 110 15325–15329. 10.1073/pnas.1307356110 24003137 PMC3780879

[B72] Mascuch S. J. Moree W. J. Hsu C.-C. Turner G. G. Cheng T. L. Blehert D. S. (2015). Direct detection of fungal siderophores on bats with white-nose syndrome via fluorescence microscopy-guided ambient ionization mass spectrometry. *PLoS One* 10:e0119668. 10.1371/journal.pone.0119668 25781976 PMC4364562

[B73] Maslo B. Fefferman N. H. (2015). A case study of bats and white-nose syndrome demonstrating how to model population viability with evolutionary effects: evolutionary rescue and PVA. *Conserv. Biol.* 29 1176–1185. 10.1111/cobi.12485 25808080

[B74] McGuire L. P. Mayberry H. W. Willis C. K. R. (2017). White-nose syndrome increases torpid metabolic rate and evaporative water loss in hibernating bats. *Am. J. Physiol. Regul. Integr. Comp. Physiol.* 313 R680–R686. 10.1152/ajpregu.00058.2017 28835446 PMC5814698

[B75] McGuire L. P. Turner J. M. Warnecke L. McGregor G. Bollinger T. K. Misra V. (2016). White-nose syndrome disease severity and a comparison of diagnostic methods. *EcoHealth* 13 60–71. 10.1007/s10393-016-1107-y 26957435

[B76] McMurdie P. J. Holmes S. (2013). phyloseq: an R package for reproducible interactive analysis and graphics of microbiome census data. *PLoS One* 8:e61217. 10.1371/journal.pone.0061217 23630581 PMC3632530

[B77] Meteyer C. U. Buckles E. L. Blehert D. S. Hicks A. C. Green D. E. Shearn-Bochsler V. (2009). Histopathologic criteria to confirm white-nose syndrome in bats. *J. Vet. Diagn. Invest.* 21 411–414. 10.1177/104063870902100401 19564488

[B78] Micalizzi E. W. Mack J. N. White G. P. Avis T. J. Smith M. L. (2017). Microbial inhibitors of the fungus *Pseudogymnoascus destructans*, the causal agent of white-nose syndrome in bats. *PLoS One* 12:e0179770. 10.1371/journal.pone.0179770 28632782 PMC5478148

[B79] Moore M. S. Field K. A. Behr M. J. Turner G. G. Furze M. E. Stern D. W. F. (2018). Energy conserving thermoregulatory patterns and lower disease severity in a bat resistant to the impacts of white-nose syndrome. *J. Comp. Physiol. B* 188 163–176. 10.1007/s00360-017-1109-2 28597237

[B80] Naik S. Bouladoux N. Wilhelm C. Molloy M. J. Salcedo R. Kastenmuller W. (2012). Compartmentalized control of skin immunity by resident commensals. *Science* 337 1115–1119. 10.1126/science.1225152 22837383 PMC3513834

[B81] Oksanen J. Blanchet F. G. Friendly M. Kindt R. Legendre P. McGlinn D. (2019). *vegan: Community Ecology Package.* Available online at: https://CRAN.R-project.org/package=vegan (accessed January 17, 2020).

[B82] Pinheiro J. Bates D. DebRoy S. Sarkar D. (2017). *R Core Team (2017) nlme: Linear and Nonlinear Mixed Effects Models. R package Version 3.1-131. Comput. Softw.* Available online at: https://cran.r-project.org/web/packages/nlme/index.html (accessed May 24, 2020).

[B83] Preheim S. P. Perrotta A. R. Friedman J. Smilie C. Brito I. Smith M. B. (2013a). Computational methods for high-throughput comparative analyses of natural microbial communities. *Methods Enzymol.* 531 353–370. 10.1016/B978-0-12-407863-5.00018-6 24060130

[B84] Preheim S. P. Perrotta A. R. Martin-Platero A. M. Gupta A. Alm E. J. (2013b). Distribution-based clustering: using ecology to refine the operational taxonomic unit. *Appl. Environ. Microbiol.* 79 6593–6603. 10.1128/AEM.00342-13 23974136 PMC3811501

[B85] Price M. N. Dehal P. S. Arkin A. P. (2010). FastTree 2 – approximately maximum-likelihood trees for large alignments. *PLoS One* 5:e9490. 10.1371/journal.pone.0009490 20224823 PMC2835736

[B86] Quast C. Pruesse E. Yilmaz P. Gerken J. Schweer T. Yarza P. (2012). The SILVA ribosomal RNA gene database project: improved data processing and web-based tools. *Nucleic Acids Res.* 41 D590–D596. 10.1093/nar/gks1219 23193283 PMC3531112

[B87] R Core Team (2018). *R: A Language and Environment for Statistical Computing; 2015.*

[B88] Råberg L. Graham A. L. Read A. F. (2009). Decomposing health: tolerance and resistance to parasites in animals. *Philos. Trans. R. Soc. B Biol. Sci.* 364 37–49. 10.1098/rstb.2008.0184 18926971 PMC2666700

[B89] Råberg L. Sim D. Read A. F. (2007). Disentangling genetic variation for resistance and tolerance to infectious diseases in animals. *Science* 318 812–814. 10.1126/science.1148526 17975068

[B90] Rebollar E. A. Bridges T. Hughey M. C. Medina D. Belden L. K. Harris R. N. (2019). Integrating the role of antifungal bacteria into skin symbiotic communities of three Neotropical frog species. *ISME J.* 13 1763–1775. 10.1038/s41396-019-0388-x 30867545 PMC6776000

[B91] Reeder D. M. Frank C. L. Turner G. G. Meteyer C. U. Kurta A. Britzke E. R. (2012). Frequent arousal from hibernation linked to severity of infection and mortality in bats with white-nose syndrome. *PLoS One* 7:e38920. 10.1371/journal.pone.0038920 22745688 PMC3380050

[B92] Reeder S. M. Palmer J. M. Prokkola J. M. Lilley T. M. Reeder D. M. Field K. A. (2017). *Pseudogymnoascus destructans* transcriptome changes during white-nose syndrome infections. *Virulence* 8 1695–1707. 10.1080/21505594.2017.1342910 28614673 PMC5810475

[B93] Reyes E. A. Bale M. J. Cannon W. H. Matsen J. M. (1981). Identification of *Pseudomonas aeruginosa* by pyocyanin production on Tech agar. *J. Clin. Microbiol.* 13 456–458. 10.1128/jcm.13.3.456-458.1981 6787067 PMC273813

[B94] Sabino-Pinto J. Bletz M. C. Islam M. M. Shimizu N. Bhuju S. Geffers R. (2016). Composition of the cutaneous bacterial community in Japanese amphibians: effects of captivity, host species, and body region. *Microb. Ecol.* 72 460–469. 10.1007/s00248-016-0797-6 27278778

[B95] Salter S. J. Cox M. J. Turek E. M. Calus S. T. Cookson W. O. Moffatt M. F. (2014). Reagent and laboratory contamination can critically impact sequence-based microbiome analyses. *BMC Biol.* 12:87. 10.1186/s12915-014-0087-z 25387460 PMC4228153

[B96] Sass G. Nazik H. Penner J. Shah H. Ansari S. R. Clemons K. V. (2017). Studies of *Pseudomonas aeruginosa* mutants indicate Pyoverdine as the central factor in inhibition of *Aspergillus fumigatus* biofilm. *J. Bacteriol.* 200:e00345-17. 10.1128/JB.00345-17 29038255 PMC5717155

[B97] Sen R. Ishak H. D. Estrada D. Dowd S. E. Hong E. Mueller U. G. (2009). Generalized antifungal activity and 454-screening of *Pseudonocardia* and *Amycolatopsis* bacteria in nests of fungus-growing ants. *Proc. Natl. Acad. Sci. U.S.A.* 106 17805–17810. 10.1073/pnas.0904827106 19805175 PMC2764928

[B98] Shannon C. E. (1948). A mathematical theory of communication. *Bell Syst. Tech. J.* 27 379–423. 10.1002/j.1538-7305.1948.tb01338.x

[B99] Svensson E. I. Råberg L. (2010). Resistance and tolerance in animal enemy–victim coevolution. *Trends Ecol. Evol.* 25 267–274. 10.1016/j.tree.2009.12.005 20092909

[B100] Trivedi J. Lachapelle J. Vanderwolf K. J. Misra V. Willis C. K. R. Ratcliffe J. M. (2017). Fungus causing white-nose syndrome in bats accumulates genetic variability in North America with no sign of recombination. *Msphere* 2:e00271-17. 10.1128/mSphereDirect.00271-17 PMC5506559 28713859

[B101] Turner G. G. Meteyer C. U. Barton H. Gumbs J. F. Reeder D. M. Overton B. (2014). Nonlethal screening of bat-wing skin with the use of ultraviolet fluorescence to detect lesions indicative of white-nose syndrome. *J. Wildl. Dis.* 50 566–573. 10.7589/2014-03-058 24854396

[B102] U.S. Geological Survey (2019). *White-nose Syndrome Occurence Map- by Year (2019). Data Last Updated 8/30/2019.* Available online at: https://s3.us-west-2.amazonaws.com/prod-is-cms-assets/wns/prod/2d8b8030- 21ac-11ea-a154-67ca1cde5e5d-WNSSpreadMap_8_30_2019.jpg (accessed April 14, 2020).

[B103] Verant M. L. Meteyer C. U. Speakman J. R. Cryan P. M. Lorch J. M. Blehert D. S. (2014). White-nose syndrome initiates a cascade of physiologic disturbances in the hibernating bat host. *BMC Physiol.* 14:10. 10.1186/s12899-014-0010-4 25487871 PMC4278231

[B104] Walke J. B. Becker M. H. Loftus S. C. House L. L. Teotonio T. L. Minbiole K. P. C. (2015). Community structure and function of amphibian skin microbes: an experiment with bullfrogs exposed to a Chytrid Fungus. *PLoS One* 10:e0139848. 10.1371/journal.pone.0139848 26445500 PMC4596541

[B105] Walter J. Maldonado-Gómez M. X. Martínez I. (2018). To engraft or not to engraft: an ecological framework for gut microbiome modulation with live microbes. *Curr. Opin. Biotechnol.* 49 129–139. 10.1016/j.copbio.2017.08.008 28866242 PMC5808858

[B106] Warnecke L. Turner J. M. Bollinger T. K. Lorch J. M. Misra V. Cryan P. M. (2012). Inoculation of bats with European *Geomyces destructans* supports the novel pathogen hypothesis for the origin of white-nose syndrome. *Proc. Natl. Acad. Sci. U.S.A.* 109 6999–7003. 10.1073/pnas.1200374109 22493237 PMC3344949

[B107] Weiss S. Xu Z. Z. Peddada S. Amir A. Bittinger K. Gonzalez A. (2017). Normalization and microbial differential abundance strategies depend upon data characteristics. *Microbiome* 5:27. 10.1186/s40168-017-0237-y 28253908 PMC5335496

[B108] Willis C. K. R. Wilcox A. (2014). Hormones and hibernation: possible links between hormone systems, winter energy balance and white-nose syndrome in bats. *Horm. Behav.* 66 66–73. 10.1016/j.yhbeh.2014.04.009 24768718

[B109] Woo V. Eshleman E. M. Rice T. Whitt J. Vallance B. A. Alenghat T. (2019). Microbiota inhibit epithelial pathogen adherence by epigenetically regulating C-type lectin expression. *Front. Immunol.* 10:928. 10.3389/fimmu.2019.00928 31134059 PMC6514056

[B110] Woodhams D. C. Brandt H. Baumgartner S. Kielgast J. Küpfer E. Tobler U. (2014). Interacting symbionts and immunity in the amphibian skin mucosome predict disease risk and probiotic effectiveness. *PLoS One* 9:e96375. 10.1371/journal.pone.0096375 24789229 PMC4005770

[B111] Wray A. K. Jusino M. A. Banik M. T. Palmer J. M. Kaarakka H. White J. P. (2018). Incidence and taxonomic richness of mosquitoes in the diets of little brown and big brown bats. *J. Mammal.* 99 668–674. 10.1093/jmammal/gyy044

[B112] Zaneveld J. R. McMinds R. Vega Thurber R. (2017). Stress and stability: applying the Anna Karenina principle to animal microbiomes. *Nat. Microbiol.* 2:17121. 10.1038/nmicrobiol.2017.121 28836573

