# Corrigendum: Antifungal Potential of the Skin Microbiota of Hibernating Big Brown Bats (Eptesicus fuscus) Infected With the Causal Agent of White-Nose Syndrome

**DOI:** 10.3389/fmicb.2020.588889

**Published:** 2020-10-26

**Authors:** Virginie Lemieux-Labonté, Nicole A. S.-Y. Dorville, Craig K. R. Willis, François-Joseph Lapointe

**Affiliations:** ^1^Département de Sciences Biologiques, Université de Montréal, Montréal, QC, Canada; ^2^Department of Biology, Centre for Forest Interdisciplinary Research, The University of Winnipeg, Winnipeg, MB, Canada

**Keywords:** fungal infection, microbiota, resistance, big brown bat, 16S rRNA gene

In the original article, there was a mistake in Figure 3 “Relative abundance of different genera in the microbiota on bat skin from before (pre-captivity) and after (post-captivity) experiment. Analysis was performed on unrarefied ASVs table of taxa with relative abundance higher or equal to 0.1%” as published. **The wrong figure version was uploaded and the pre-captivity and post-captivity labels were inverted**. The corrected Figure 3 appears below.

**Figure 3:**
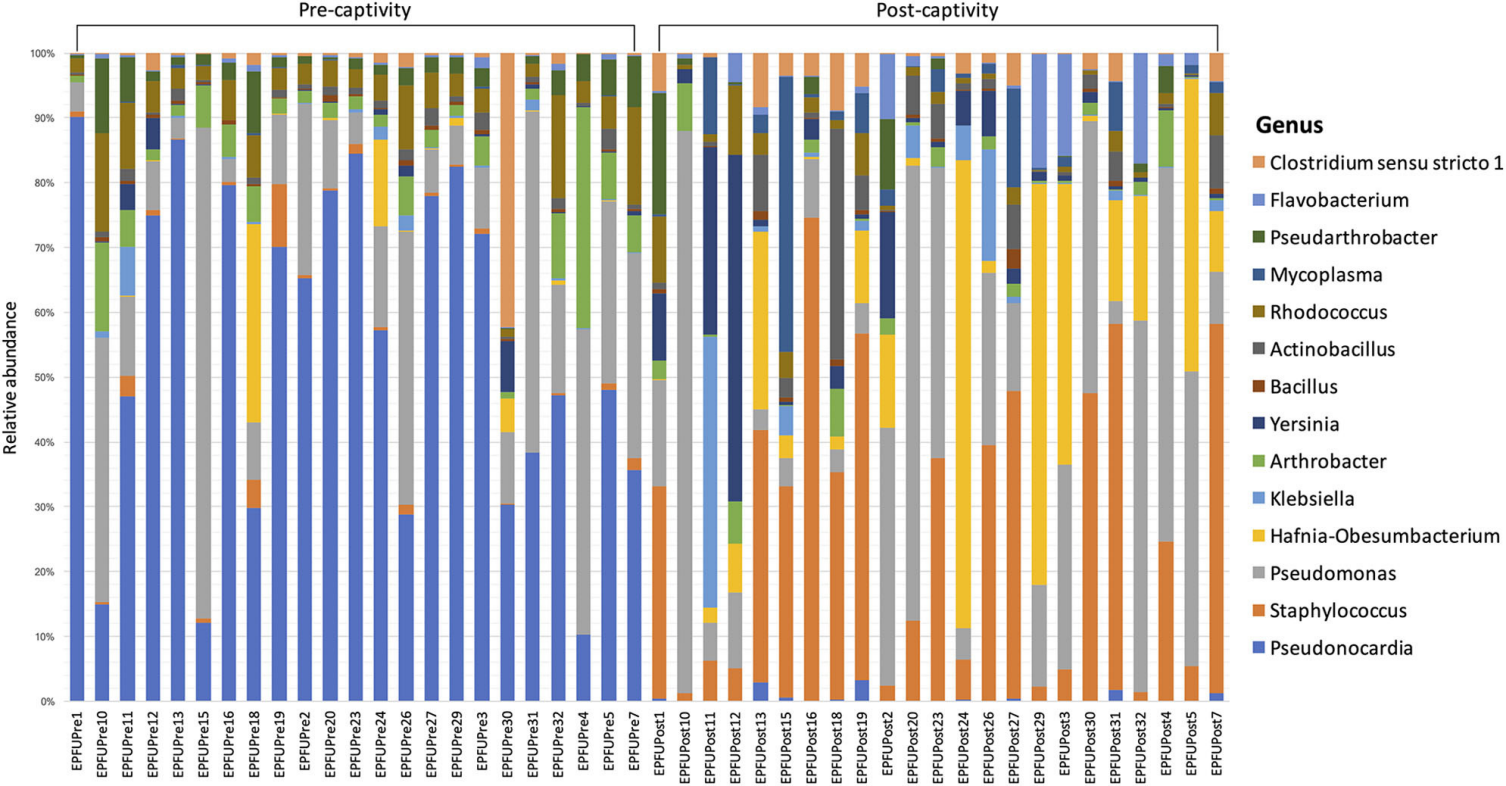
Relative abundance of different genera in the microbiota on bat skin from before (pre-captivity) and after (post-captivity) experiment. Analysis was performed on unrarefied ASVs table of taxa with relative abundance higher or equal to 0.1%.

The authors apologize for this error and state that this does not change the scientific conclusions of the article in any way. The original article has been updated.

