# Supplementary material for: Antifungal Potential of the Skin Microbiota of Hibernating Big Brown Bats (Eptesicus fuscus) Infected With the Causal Agent of White-Nose Syndrome
Source: Front Microbiol. 2020 Jul 23;11:1776. doi: 10.3389/fmicb.2020.01776 (PMC7390961; doi:10.3389/fmicb.2020.01776)
Supplement: Supplementary file 1 [file Data_Sheet_1.zip › Supplementary_files_Revised_Frontier/Supplementary_file_7.docx]

Supplementary file 6. C_t_score, *Pd*load and UV proportions of *E. fuscus* bats.

| SampleID | Inoculation^1^ | Pre Ct score | Pre *Pd* load  (ng) | Post Ct score | Post *Pd* load  (ng) | Orange UV proportion (%) |
| --- | --- | --- | --- | --- | --- | --- |
| EPFU11 | PBST | >41 | 0 | 36.30303955 | 0.0000553 | 0 |
| EPFU13 | PBST | >41 | 0 | >41 | 0 | 0 |
| EPFU15 | PBST | >41 | 0 | 37.04833603 | 0.0000331 | 0 |
| EPFU18 | PBST | >41 | 0 | 36.60210991 | 0.000045 | 0 |
| EPFU19 | PBST | >41 | 0 | >41 | 0 | 0 |
| EPFU24 | PBST | >41 | 0 | >41 | 0 | 0 |
| EPFU26 | PBST | 39.72719574 | 0.0000052 | 36.17507362 | 0.0000604 | 0.072 |
| EPFU30 | PBST | 39.73607567 | 0.0000052 | 37.16534805 | 0.0000305 | 0.016 |
| EPFU31 | PBST | 39.55136335 | 0.0000059 | >41 | 0 | 0.077 |
| EPFU4 | PBST | 36.75018501 | 0.0000406 | 33.5191288 | 0.000375 | 1.092 |
| EPFU7 | PBST | >41 | 0 | 39.70586777 | 0.0000053 | 0.003 |
| EPFU1 | *Pd* | >41 | 0 | 33.46580124 | 0.000389 | 8.416 |
| EPFU10 | *Pd* | 39.52378273 | 0.000006 | 32.56645966 | 0.0007221 | 6.758 |
| EPFU12 | *Pd* | >41 | 0 | 35.96902313 | 0.0000695 | 1.785 |
| EPFU16 | *Pd* | >41 | 0 | 35.51846504 | 0.0000948 | 0.203 |
| EPFU2 | *Pd* | >41 | 0 | 32.42738152 | 0.0007946 | 6.956 |
| EPFU20 | *Pd* | >41 | 0 | 34.37360573 | 0.0002084 | 0.731 |
| EPFU23 | *Pd* | 39.36528415 | 0.0000067 | 34.9600029 | 0.0001392 | 1.35 |
| EPFU27 | *Pd* | >41 | 0 | 38.65805817 | 0.0000109 | 0.517 |
| EPFU29 | *Pd* | >41 | 0 | 36.61650467 | 0.0000446 | 0.121 |
| EPFU3 | *Pd* | 39.60971569 | 0.0000057 | 36.68639946 | 0.0000425 | 0.444 |
| EPFU32 | *Pd* | 39.76990642 | 0.0000051 | 32.7359333 | 0.0006427 | 6.682 |
| EPFU5 | *Pd* | >41 | 0 | 38.81292343 | 0.0000098 | 0.237 |

^1.Pd:^ *^Pd^* ^inoculated bats, PBST: sham inoculated bats PBS + 0.5%Tween 20^
