# Supplementary material for: Antifungal Potential of the Skin Microbiota of Hibernating Big Brown Bats (Eptesicus fuscus) Infected With the Causal Agent of White-Nose Syndrome
Source: Front Microbiol. 2020 Jul 23;11:1776. doi: 10.3389/fmicb.2020.01776 (PMC7390961; doi:10.3389/fmicb.2020.01776)
Supplement: Supplementary file 1 [file Data_Sheet_1.zip › Supplementary_files_Revised_Frontier/Supplementary_file_1.docx]

**Supplementary file 1. Shannon diversity of *E. fuscus* skin microbiota samples tested by Anova of linear model.** Transport model compare 8 bats skin microbiota sampled in the cave at capture with bats sampled in lab less than 24h later.

| **Model** | **F statistic** | ***P*-value** |
| --- | --- | --- |
| Transport | 3.36 | 0.08 |

Significant results are in bold.
