# Supplementary material for: Antifungal Potential of the Skin Microbiota of Hibernating Big Brown Bats (Eptesicus fuscus) Infected With the Causal Agent of White-Nose Syndrome
Source: Front Microbiol. 2020 Jul 23;11:1776. doi: 10.3389/fmicb.2020.01776 (PMC7390961; doi:10.3389/fmicb.2020.01776)
Supplement: Supplementary file 1 [file Data_Sheet_1.zip › Supplementary_files_Revised_Frontier/Supplementary_file_3.docx]

Supplementary file 3. Rectal temperature record of *E. fuscus* at the end of captivity.

| SampleID | Inoculation^1^ | Time Exit Incubator | Time Rectal Temperature | Rectal Temperature (°C) |
| --- | --- | --- | --- | --- |
| EPFU11 | PBST | 12:15 | 12:17 | 10 |
| EPFU13 | PBST | 11:47 | 11:49 | 9.2 |
| EPFU15 | PBST | 11:42 | 11:45 | 9.2 |
| EPFU18 | PBST | 12:34 | 12:35 | 9.9 |
| EPFU19 | PBST | 14:10 | 14:10 | 16.7 |
| EPFU24 | PBST | 14:14 | 14:14 | 29 |
| EPFU26 | PBST | 14:47 | 14:48 | 13.2 |
| EPFU30 | PBST | 12:24 | 12:24 | 13.5 |
| EPFU31 | PBST | 11:33 | 11:35 | 10.2 |
| EPFU4 | PBST | 14:53 | 14:54 | 16.3 |
| EPFU7 | PBST | 14:03 | 14:03 | 13.3 |
| EPFU1 | *Pd* | 15:26 | 15:26 | 11.3 |
| EPFU10 | *Pd* | 15:47 | 15:48 | 33.3 |
| EPFU12 | *Pd* | 11:40 | 11:41 | 10.1 |
| EPFU16 | *Pd* | 15:19 | 12:17 | 10.2 |
| EPFU2 | *Pd* | 15:50 | 15:50 | 15.7 |
| EPFU20 | *Pd* | 15:42 | 15:43 | 30.5 |
| EPFU23 | *Pd* | 15:34 | 15:35 | 15.9 |
| EPFU27 | *Pd* | 12:11 | 12:12 | 10.9 |
| EPFU29 | *Pd* | 11:46 | 11:47 | 35.3 |
| EPFU3 | *Pd* | 15:46 | 15:46 | 35.1 |
| EPFU32 | *Pd* | 15:39 | 15:40 | 14.4 |
| EPFU5 | *Pd* | 11:34 | 11:35 | 12.7 |

^1.Pd:^ *^Pd^* ^inoculated bats, PBST: sham inoculated bats PBS + 0.5%Tween 20^
