# Supplementary material for: Antifungal Potential of the Skin Microbiota of Hibernating Big Brown Bats (Eptesicus fuscus) Infected With the Causal Agent of White-Nose Syndrome
Source: Front Microbiol. 2020 Jul 23;11:1776. doi: 10.3389/fmicb.2020.01776 (PMC7390961; doi:10.3389/fmicb.2020.01776)
Supplement: Supplementary file 1 [file Data_Sheet_1.zip › Supplementary_files_Revised_Frontier/Supplementary file captions.docx]

Supplementary file 1. Shannon diversity of *E. fuscus* skin microbiota samples tested by Anova of linear model. Transport model compare 8 bats skin microbiota sampled in the cave at capture with bats sampled in lab less than 24h later.

Supplementary file 2. db-RDA of unweighted and weighted UniFrac distances of *E. fuscus* skin microbiota samples. Transport model compare 8 bats skin microbiota sampled in the cave at capture with bats sampled in lab less than 24h later. Sex factor was controlled.

Supplementary file 3. Rectal temperature record of *E. fuscus* at the end of captivity.

Supplementary file 4. Principal coordinates analysis of square rooted weighted UniFrac distances. Each point represents a control, mock, or individual bat sample.

Supplementary file 5. Relative abundance of different genera in the Mock sample. The analysis was performed on unrarefied ASVs table of taxa with relative abundances higher or equal to 0.1%.

Supplementary file 6. Rarefaction curves of alpha diversity calculated on multiple rarefied data for each of the 46 pre-(blue) and post-captive(coral) bat skin microbiota samples. Left panel presents overall richness (OTUs observed) and right panel presents Shannon diversity of bat skin samples.

Supplementary file 7. C_t_score, *Pd*load and UV proportions of *E. fuscus* bats.
